# VANET Clustering Based Routing Protocol Suitable for Deserts

**DOI:** 10.3390/s16040478

**Published:** 2016-04-06

**Authors:** Mohammed Mohsen Mohammed Nasr, Abdeldime Mohamed Salih Abdelgader, Zhi-Gong Wang, Lian-Feng Shen

**Affiliations:** 1 National Mobile Communications Research Laboratory, Southeast University, Nanjing 210096, China; 2 School of Information Science and Engineering, Southeast University, Nanjing 210096, China; abdeldime@hotmail.com (A.M.S.A.); zgwang@seu.edu.cn (Z.-G.W.); 3 Electrical and Computer Department, College of Engineering, Karary University, Khartoum 12304, Sudan

**Keywords:** *ad hoc* networks, VANET, clustering, routing, CBRP

## Abstract

In recent years, there has emerged applications of vehicular *ad hoc* networks (VANETs) towards security, safety, rescue, exploration, military and communication redundancy systems in non-populated areas, besides its ordinary use in urban environments as an essential part of intelligent transportation systems (ITS). This paper proposes a novel algorithm for the process of organizing a cluster structure and cluster head election (CHE) suitable for VANETs. Moreover, it presents a robust clustering-based routing protocol, which is appropriate for deserts and can achieve high communication efficiency, ensuring reliable information delivery and optimal exploitation of the equipment on each vehicle. A comprehensive simulation is conducted to evaluate the performance of the proposed CHE and routing algorithms.

## 1. Introduction

A vehicular *ad hoc* network (VANET) primarily serves as a mesh network consisting of mobile and fixed nodes. It is a form of mobile *ad hoc* network (MANET) used to provide communication among the adjacent vehicles and the nearby fixed equipment. It is defined as a network without infrastructure and centralized administration [1,2,3]. Intelligent vehicular ad hoc networking (IVANET) provides an intelligent way of using vehicular networking [4]. It targets supporting vehicular safety, traffic monitoring, accident prevention and many other applications. Although VANET is a subclass of MANET having different features from other types of *ad hoc* networks, such as wireless sensor networks (WSNs) and delay-tolerant networks (DTNs), the nodes in VANETs can be equipped to serve as a host and router at the same time [5]. This special property and capability qualifies VANETs to form and set up a network connection in extraordinary environments, such as deserts, forests, mountains and in natural disaster situations, where ordinary communication infrastructure is lacking. Furthermore, the dynamic topological characteristics enable VANETs either to work as a standalone network or as a part of public networks, such as the Internet, using satellites and other communication links.

Recently, economic development and the popularization of automobiles have brought high concern to VANETs. In 2003, the Federal Communications Commission (FCC) developed the Dedicated Short-Range Communications (DSRC) standard for dynamic organizing vehicular networking communication, which provides communications between the on board units (OBUs) of the vehicle and the roadside units (RSUs) in specific locations, as well as supporting particular ITS applications. DSRC operates on radio frequencies in the 5.85 GHz to 5.925 GHz range, which is the standard Industrial, Scientific and Medical (ISM) band. In 2009, the IEEE 802.11 standard was developed to launch a new standard for vehicle communication, named IEEE 802.11p [6,7,8,9,10,11].

We noticed that previous researchers generally concentrated on VANETs located in cities or on highways. However, with economic development and from environmental perspectives, people’s activities gradually have extended to rugged environments, such as deserts, forest and other unpopulated areas. Taking into account the irregular road conditions and lack of ordinary communication facilities in these environments, safe driving of a vehicle is quite challenging and even fatal. Although satellite and mobile communication can partially solve the problem of information exchange between vehicles, they are rather expensive and have limited bandwidth. Moreover, mobile communication systems covering such extraordinary regions are sometimes technically difficult. Therefore, a VANET is possibly a great alternative solution.

In VANETs, topology-based routing protocols use link information that exists in the network to perform packet forwarding. They are divided into proactive and reactive protocols. The reactive one usually opens the route only when it is necessary for a node to communicate with another. It maintains only the routes that are currently in use. Therefore, it reduces the burden in the network. It consists of a route discovery phase in which the query packets are flooded into the network for the path search, and this phase completes when the route is found. The common sorts of reactive routing protocols in V2V communication are Ad hoc On-demand Distance Vector Routing (AODV), Temporally Ordered Routing Algorithm (TORA) and dedicated short-range (DSR) [12,13]. Another type of V2V routing protocol is cluster-based routing protocols in which a group of nodes identifies themselves to be a part of a cluster, and a node designated as the CH will organize and broadcast the packet to the cluster. Reasonable scalability can be provided for large networks; however, network delays and overhead are incurred when forming clusters in highly mobile VANETs. In cluster-based routing, virtual network infrastructure must be created through the clustering of nodes in order to provide scalability. The common types of cluster-based routing protocols are cluster base routing protocol (CBRP), Clustering for Open IVC (*Inter-vehicle communication*) Network (COIN) and Location Routing Algorithm with Cluster- Based Flooding (LORA-CBF) [12,13,14,15]. However, considering the high mobility of vehicles, irregular roads and environmental factors, the traditional MANET and VANET routing protocols may not satisfy network communication and information exchange in these environments. Therefore, clustering routing protocols became one of the most important choices.

This paper presents a VANET clustering routing algorithm appropriate for desert and other rugged environments. The proposed protocol provides a stable clustering structure and a reliable route between the source and destination vehicles. Additionally, it utilizes vehicles equipped with mobile or satellite links to act as a gateway for unreachable destinations. The cluster structure, cluster head election (CHE) and the routing protocol are presented and comprehensively described.

A new CHE approach is also proposed and theoretically analyzed. Throughout the paper, numerical simulation is used to evaluate the proposed CHE approach. Simulation results are also conducted to evaluate the proposed routing algorithm, in terms of the packet delivery ration (PDR), end-to-end delay and cluster stability, and to compare it to other alternatives.

The rest of the paper is organized as follows. Section 2 briefly introduces the most important related works. Section 3 describes the cluster structure of the general designed model of the network and its corresponding logical model. Section 4 describes the clustering process and presents the CHE procedure. Section 5 explains and extensively discusses the proposed routing protocol. Section 6 is the simulation results and observations. Finally, Section 7 concludes this work.

## 2. Related Work

Due to the reliability of its information transmission and wide range of applications, vehicle dynamic organizing networks have emerged with a high degree of concern for both the industrial and research aspects. Considerable research has explored communication algorithms to satisfy the special features of VANETs. For instance, Yu *et al*. [1] used a distributed learning algorithm to dynamically change the transmission rate between adjacent vehicles. By this algorithm, each vehicle learns from local observations and selects a delay based on learning results, so that data can be received efficiently and aggregated. Chin *et al*. [2] designed and implemented two types of routing protocols and compared them to protocols representing MANETs and VANETs. Many other reports stated an overview of highway cooperative collision avoidance (CCA), which is an emerging vehicular safety application using the IEEE-802.11 and DSRC standards [7,16].

Vehicles’ movement direction is quite important in determining the quality of the communication system in VANETs and has a great significance in restricting the routing protocols’ capabilities and performance. Therefore, many researchers have given considerable attention to the vehicle movement trends. In this context, Zhang *et al.* [3] considered the effect of the driving behaviors and vehicle classification in the movement direction and consequently incorporated these effects into the route-finding process. Their proposed protocol, as an energy-efficient routing protocol, classifies the vehicles into several categories and then examines the effect of the vehicle movement trends to aid in making a routing decision. It predicts the movement direction using the current directions and the next directions after going through the road intersections. Although it demonstrated reasonable results in terms of energy consumption in urban scenarios, it greatly relies on the road intersection while performing the routing process.

Wang *et al.* proposed a vehicular mobility model that reflects real-world vehicle movement while studying the performance of packet-routing protocols in small-scale and large-scale VANETs. Furthermore, the connection-based restricted forwarding (CBRF) and connection-less geographic forwarding (CLGF) algorithms were presented according to the environment, quantity and speed of the vehicles. These algorithms were employed to determine the shortest communication distance *r*; subsequently, *r* was used to determine the shortest route [8]. Zhang *et al.* [9] proposed an analytical model to predict both up-link and down-link connectivity probabilities along with deriving the urban environment route by means of roadside auxiliary facilities. Others proposed a broadcasting routing protocol, addressing both the broadcast storm and connected network problems in urban VANETs where both direct and indirect packet routing protocols were utilized [10].

In addition to safety concerns, VANETs can also support other applications, which require guaranteed quality of service (QoS), particularly in the case of multimedia and data transfer applications. In this context, a routing protocol for VANETs called the intersection-based geographical routing protocol (IGRP) was proposed [4]. IGRP is based on an effective selection of road intersections through which a packet must pass to reach the gateway to the network. The selection is made in such way that it can guarantee high probability, bandwidth utilization, error rate optimization and network connectivity among the road intersections, while satisfying QoS. Geographical forwarding is used to transfer packets between any two intersections on the path, reducing the route’s sensitivity to the individual node movements [4]. Other methods use urban roadside infrastructure instead of road intersections. In this methods, vehicles regularly send messages to the roadside infrastructure, which is responsible for the routing process [5,14,17].

Due to continuous expansion of VANETs’ structure, clustering is a popular means of organizing networks in MANETs and VANET. Consequently, clustering routing algorithms attracted many researchers, in favor of its convenience and useful features. Many clustering solutions, including topology-based clustering, mobility-based clustering, identifier neighbor-based clustering, energy-based clustering and weight-based clustering, have been proposed [7,18,19,20,21,22]. In urban environments, the integration of VANET with 3G networks was investigated, where a VANET is divided into different clusters according to the movement direction of the vehicle, velocity, signal strength or VANET coverage range. The nearest vehicle to the center of the cluster is dynamically selected as a cluster head (CH). When a cluster member requires external communication, the selection of the gateway is based on the speed of the CH, signal strength and communication connectivity [4]. Sun *et al.* [17] presented an analytical design-based simulation of environmentally-aware clustering algorithms for wireless *ad hoc* networks. The clustering algorithm establishes an adaptive dynamic arrangement that enables multi-mode routing scenarios needed to enhance scalability and robustness. In this method, clustering is based on a criterion that enforces an upper bound on the probability of path failure within the cluster over time. Moreover, Li *et al.* [6] introduced a clustering technique suitable for a VANET environment on highways with the aim of enhancing the stability of the network topology. This technique takes the speed difference a parameter for creating a relatively stable cluster structure. It also developed a new multi-metric algorithm for CHE.

One of the popular clustering algorithms is the affinity propagation (AP) algorithm, which has been recently proposed [23]. AP is a distance-based clustering algorithm, which results in the frequent changing of CHs when speed effectively changes. Moreover, AP requires a number of iterative loops that increase the delay time of cluster construction. To adapt the new features of VANET, a modified distributed mobility adaptive clustering algorithm has been presented in [24]. Since this modified algorithm is mobility adaptive, distributed and traffic direction dependent, re-clustering is avoided when the vehicles move in different directions.

Some other literature works proposed a cluster-based directional routing protocol (CBDRP) suitable for highway scenarios in which the CH selects another one according to the direction of motion in order to forward packets [14]. In the cluster-based routing protocol (CBRP), VANETs are characterized by high vehicle mobility. Due to high mobility, the VANET topology changes rapidly, resulting in high communication overhead for exchanging new topological information. For more information, the readers are invited to review most of the well-known clustering solutions for VANETs in [15].

Most of the aforementioned algorithms are based on one-hop clustering, which only allows communication among one-hop neighbors. Consequently, the coverage rang is small, and many clusters are formed, which decreases cluster stability. Clustering algorithms are often designed to assure cluster stability, which is essential to reduce the maintenance cost and increase routing efficiency and throughput. In one-hop clustering algorithms, vehicles must identify the aggregate mobility of all *M* hops-distant neighbors. Consequently, various extra control messages are produced and broadcast within the network, which ultimately reduces the efficiency of cluster formation. Moreover, the majority of these research works only considered the location or the speed of the vehicles for constructing the clusters, while the direction or the equipment of the vehicles have not been thoroughly considered. These protocols also used various strategies having different limitations and drawbacks when selecting the CH. Most importantly, all of these protocols and solutions have never been investigated in desert and rugged scenarios. The majority of these protocols rely on road side units, road intersections or other assumptions, which may not satisfy desert scenarios.

## 3. System Model and Network Logical Structure

**Figure 1:**
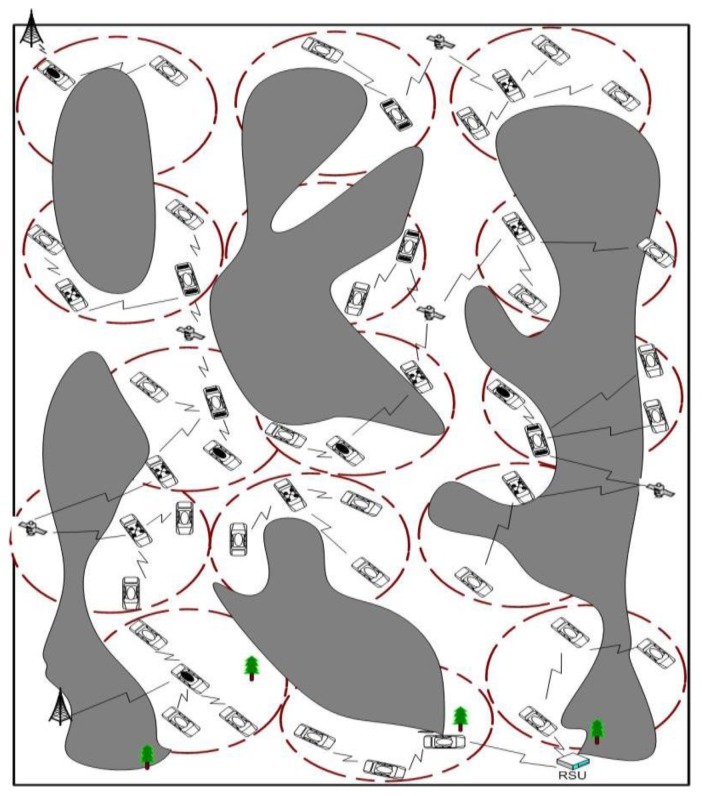
System model.

The VANET scenarios adopted throughout this paper are in desert environments without ordinary communication facilities. This work considers a group of vehicles working on a specific mission. The proposed system model assumes the number of vehicles (N), divided into different clusters according to their location, as shown in Figure 1. Each cluster (C) has one CH and cluster members (Cm). The number of vehicles within one cluster is $N_{c}$ and the maximum number of vehicles for one cluster $N_{max}$. The number of hops is *M*, and the number of maximum allowable hops is $M_{max}$. The primary communication distance of the normal VANET for two vehicles *i*,*j* is *L*, which can be expressed as:

(1) $$L=\sqrt{{\left(x_{i}-x_{j}\right)}^{2}+{\left(y_{i}-y_{j}\right)}^{2}}$$

Here, *x*, *y* represent the coordinates of the vehicles *i*, *j* and $L\leq L_{vmax}$, where $L_{vmax}$ is the maximum communication distance between two vehicles that is determined according the PHY specification of the communication system. However, this communication distance can be extended using the multi-hop property of VANETs. This means, the maximum distance $L_{max}$ can be obtained by:

(2) $$L_{max}=M_{max}L_{vmax}$$

For a specific number of hops *M* using $K\leq N_{c}$ vehicles, the communication distance can be obtained as:

(3) $$L_{M}=\sum_{m=1}^{M}L_{m}$$

Here, $L_{m}$ represents the communication distance of a specific hop *m*.

The direction, the location and speed information of the vehicles can be collected from the GPS of each vehicle. The vehicle periodically broadcasts this information using the *ad hoc* communication. Vehicles within the coverage region store this information and forward it to the next hop together with their information. After a number of transmissions, each vehicle has the information of the other vehicles. In this paper, we assume that the vehicles are firstly classified into four groups according to their directions. We employed C4.5 [25] as a simple classification tree algorithm to classify the vehicles into four groups using a simple training set shown in Table 1. For each group, the clustering operation is performed, and the CH is accordingly elected.

**Figure 2:**
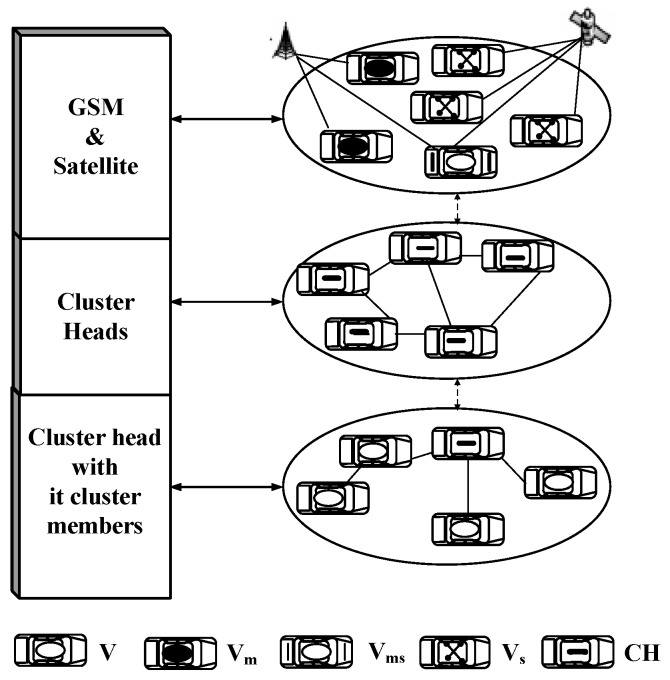
Logical structure model.

The designed model can be logically represented by three layers, which form the logical structure of the network, as shown in Figure 2. The bottom layer describes the communication network of the CH with its cluster members and the internal communication within the cluster. The middle layer, which acts as a network backbone, describes the communication between the cluster’s heads. The upper layer is formed by the vehicles equipped with satellite and mobile links to outside networks.

## 4. Clustering and Cluster Management

**Figure 3:**
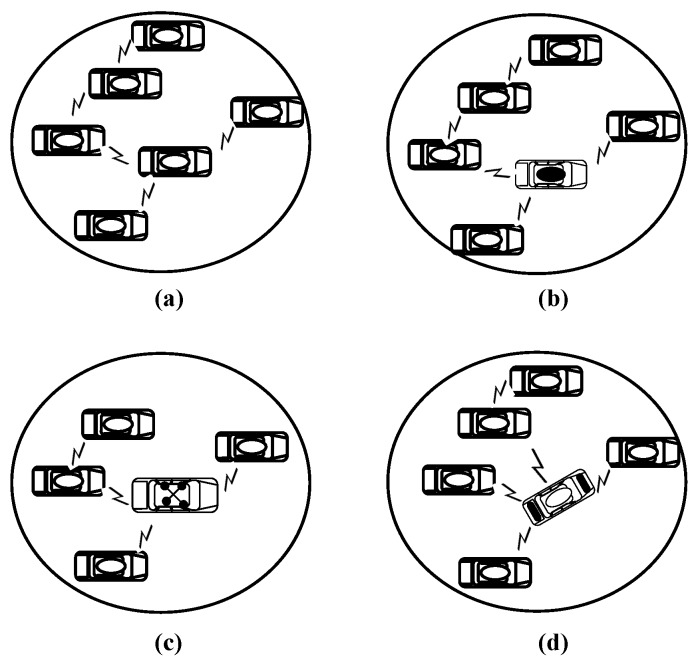
The main four clusters’ structures. (**a**) Cluster type (V), (**b**) Cluster type $\left(V,V_{m}\right)$, (**c**) Cluster type $\left(V,V_{s}\right)$ and (**d**) Cluster type $\left(V,V_{ms}\right)$.

In this paper, a cluster is the basis of the designed model. After considering the direction of the vehicles, this paper classified vehicles into four categories depending on the communication equipment. These categories are the main factor in clustering classification and play an important role in cluster composition and CHE. The four categories include the vehicles equipped with VANET communication only, vehicles equipped with mobile communication, vehicles equipped with satellite communication and vehicles equipped with both mobile and satellite communication. We used the notation *V*, $V_{m}$, $V_{s}$ and $V_{ms}$ to represent the aforementioned four categories, respectively. The movement of the vehicles dynamically yields different types of clusters. However, this paper only considers the four main clusters shown in Figure 3. The first cluster type consists of *V* only, as in Figure 3a; the second type contains both *V* and $V_{m}$, as in Figure 3b; the third type consists of both *V* and $V_{s}$, as shown in Figure 3c; and the fourth type is a combination of *V* and $V_{ms}$, as shown in the Figure 3d. All of them are able to communicate with the outside network, except the first type.

**Figure 4:**
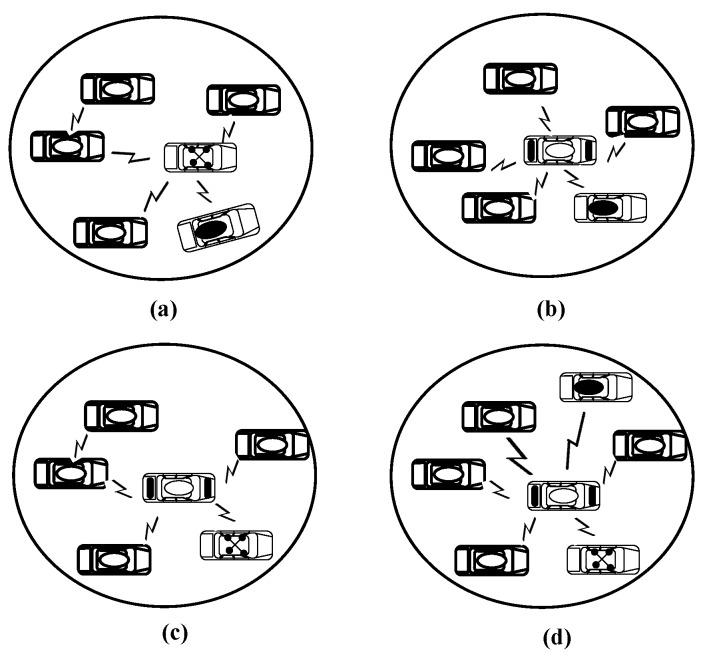
Samples of other cluster structures. (**a**) Cluster type $\left(V,V_{m},V_{s}\right)$; (**b**) Cluster type $\left(V,V_{m},V_{ms}\right)$; (**c**) Cluster type $\left(V,V_{s},V_{ms}\right)$ and (**d**) Cluster type $\left(V,V_{m},V_{s},V_{ms}\right)$.

Figure 4 shows a sample of possible other types of clusters, which can be formed according to the vehicle’s categories. However, the main four types in Figure 3 can represent all. A CH should be elected for each type of cluster. For the first type, the CH should be elected according to the location, and velocity. Other types will follow the election procedure described in Section 4.2.

According to the logical structure, the CH and the neighboring CH can communicate directly; however, in the actual situations, the CH and the neighboring CHs are not necessarily located within the same VANET communication range. For that reason, the CH may select one of the boundary cluster members to act as a gateway for forwarding traffic information (both control and data messages) between the neighboring CHs. After the completion of the CHE process, each CH collects and stores the location, direction, velocity and equipment information of its cluster members; later, it uses this information to calculate the routes.

When a cluster member requests a communication, it will send a request message to its CH. The CH checks whether the destination is within its cluster members or not. If the destination is one of its cluster members, it will directly forward the requested route information to the source member. In case the destination vehicle is located in another cluster, the CH, directly or using adjacent cluster members as a gateway, forwards the route information to the destination CH. The destination CH returns the requested route information to the source CH. Finally, the source CH sends the route information to its intended cluster member. Therefore, the clustering process is not only the election of the, CH but also includes the operation of determining boundary vehicles used to communicate between the adjacent clusters.

**Figure 5:**
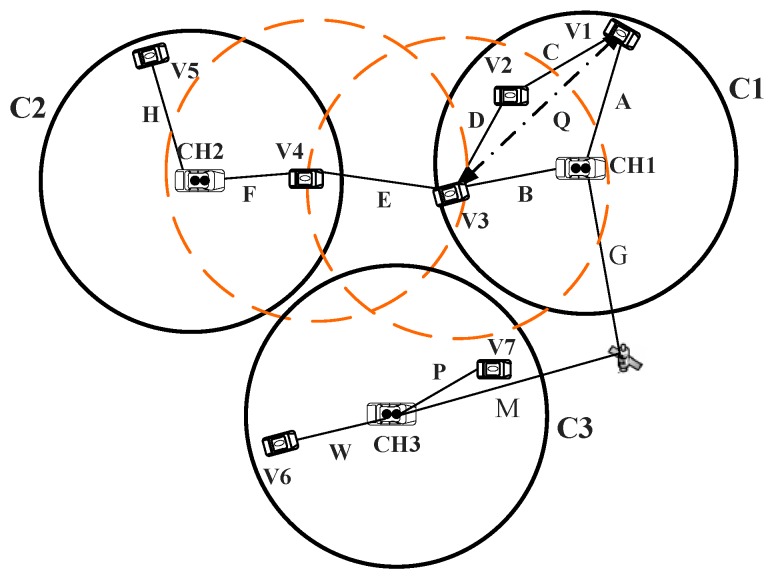
Communication within a cluster and between adjacent clusters.

In Figure 5, we assume three clusters $C_{1}$, $C_{2}$ and $C_{3}$ with their clusters heads $CH_{1}$, $CH_{2}$ and $CH_{3}$, respectively. As the distance between $CH_{1}$ and $CH_{2}$ is beyond the coverage of the VANET, the CHs can use the adjacent cluster members $V_{3}$ and $V_{4}$ to communicate with each other, because these two vehicles are within the same VANET coverage; however, they belong to different clusters. The communication between $CH_{1}$ and $CH_{3}$ will be through a mobile or satellite solution, because not only the distance between them is out of the coverage area, but also the distance between the adjacent neighbors is out of the VANET communication coverage. The CHs usually provide the communication between their cluster members. For public use, they can offer this service with some fees. However, for a group of vehicles working on a specific mission, such as a rescue, mining and military missions, the CH would provide this service for free.

It is very important to take into consideration that the increase of cluster members will increase the burden on the CH. Hence, the intra-cluster communication cost will increase, because communication between the vehicles and CH, as well as the neighboring CHs will increase the number of hops. Therefore, this paper restricts the maximum number of hops to $M_{max}$.

### 4.1. Cluster Head Election Principles

A CH is responsible for passing packets and organizing inter-/intra-cluster information traffic. The perfect election of the cluster will offer an efficient network performance and high reliability. Most of the clustering routing protocols normally elect the CH based on the location or velocity. On the one hand, the selection using location is not usually an optimum choice, because the location of the selected vehicle may rapidly change according to the relative speed with the other vehicles in the network. This means that the vehicle with the best location may quickly become unsuitable as the CH. On the other hand, selecting the CH according to the velocity is also not appropriate in many situations, particularly when the relative movement between vehicles is very high. This will consequently lead to cluster instability. Moreover, the speed of the vehicles directly affects their locations. Therefore, the perfect CH selection method should consider many factors to obtain high cluster stability and network reliability. In this paper, we proposes a new approach to select the CH by considering the location, velocity and the equipment of the vehicles in order to obtain a high cluster stability and for optimum utilization of available equipment in the network, particularly in the case of vehicles working on a specific mission.

The CH is elected from the vehicles traveling in the same direction. The notation *P* represents the CHE priority factor. For a specific cluster, the election priority factor *P* is calculated according to the equipment, location and velocity of the vehicle. For the CHE process purposes, we assume the following:

1. Each vehicle has a unique ID number.
2. Each vehicle is equipped with a satellite positioning device (GPS) used to collect location information and to periodically obtain velocity and direction information.
3. In the proposed model, *υ* is the instantaneous velocity of each vehicle.
4. The symbol *i* represents the intended vehicle, $\phi_{n}$ for the group of vehicles in the same cluster, $\phi_{i}$ for the group of vehicles neighboring vehicle *i*, and *j* is another vehicle in the same cluster. Each vehicle has $N_{c}-1$ neighbors, $\left[j_{1},j_{2},...,j_{n-1}\right]$.

The calculation of *P* is as follows.

#### 4.1.1. Vehicle Equipment Priority

According to the four types of vehicles, we assume that the vehicle equipment priority factor value $P_{e}$ is as follows:

(4) $$P_{e}=\left[\begin{array}{ccccc} 3 & for & vehicles & type & V\\ 2 & for & vehicles & type & V_{s}\\ 1 & for & vehicles & type & V_{m}\\ 0 & for & vehicles & type & V_{ms} \end{array}\right.$$

For one cluster, the equipment priority of the vehicles can be obtained by:

(5) $$P_{e_{I}}=\left[\begin{array}{ccccc} P_{e_{1}} & P_{e_{2}} & P_{e_{3}} & ... & P_{e_{N_{c}}} \end{array}\right]$$

These values can be normalized by scaling them between zero and one as:

(6) $$P_{e_{norm}}=\frac{P_{e}\left(i\right)-min\left(P_{e_{I}}\right)}{max\left(P_{e_{I}}\right)-min\left(P_{e_{I}}\right)}\;\;\;\;\forall i\in \phi_{n}$$

The vehicle with the least $P_{e_{norm}}$ value has the highest equipment priority.

#### 4.1.2. Location Priority

The distances between *i* and *j* are obtained by:

(7) $$D_{ij}=\sqrt{{\left(x_{i}-x_{j}\right)}^{2}+{\left(y_{i}-y_{j}\right)}^{2}}\;\;\;\forall j\in \phi_{i}$$

Each vehicle has $\left(N_{c}-1\right)$ distance values $\left(D_{ij}\right)$, and the summation of all $\left(D_{ij}\right)$ values of the vehicle (i) is obtained by:

(8) $$S_{d}=\sum_{j=1}^{N_{c}-1}D_{ij}$$

A vehicle with least $S_{d}$ is the nearest one to all other vehicles. It will take a higher priority weight in the CH location priority election. It is not important to be in the center of the cluster, but it is the nearest one to others. In addition, it is not important to be elected as a CH, because the election not only depends on location, but also depends on other parameters. Therefore, another notation is the location priority factor $P_{l}$, which is used when there are additional priority metrics, such as equipment priority, ID priority and velocity priority, to calculate the over-all priority of a certain vehicle. The value of $P_{l}$ is used to represent the location priority deviation of a vehicle with respect to the vehicle of the lower $S_{d}$. The following equations are used to calculate $P_{l}$:

(9) $$S_{\mu }=min\left[S_{d}\left(1\right),S_{d}\left(2\right),S_{d}\left(3\right),...,S_{d}\left(N_{c}\right)\right]$$

(10) $$P_{l}=S_{d}\left(i\right)-S_{\mu }\;\;\;\forall i\in \phi_{n}$$

For one cluster, the location priority of the vehicles can be obtained by:

(11) $$P_{l_{I}}=\left[\begin{array}{ccccc} P_{l_{1}} & P_{l_{2}} & P_{l_{3}} & ... & P_{l_{N_{c}}} \end{array}\right]$$

These values can be normalized by scaling them between zero and one as:

(12) $$P_{l_{norm}}=\frac{P_{l}\left(i\right)-min\left(P_{l_{I}}\right)}{max\left(P_{l_{I}}\right)-min\left(P_{l_{I}}\right)}\;\;\;\;\forall i\in \phi_{n}$$

For the vehicle with less $S_{d}$, $P_{l_{norm}}=0$.

#### 4.1.3. Vehicle Velocity Priority

We assume: $\upsilon_{1}$, $\upsilon_{2}$, $\upsilon_{3}$ ... $\upsilon_{n}$ are the instantaneous velocities of the vehicles and $\upsilon_{\mu }$ is the mean velocity at time instant *t*. Calculate $P_{\upsilon }$ for each vehicle as follows:

(13) $$P_{v}=\frac{1}{n}\left(\upsilon \left(i\right)-\upsilon_{\mu }\right)\;\;\;\forall i\in \phi_{n}$$

For one cluster, the velocity priority of the vehicles can be obtained by:

(14) $$P_{\upsilon_{I}}=\left[\begin{array}{ccccc} P_{\upsilon_{1}} & P_{\upsilon_{2}} & P_{\upsilon_{3}} & ... & P_{\upsilon_{N_{c}}} \end{array}\right]$$

These values can be normalized by scaling them between zero and one as:

(15) $$P_{\upsilon_{norm}}=\frac{P_{\upsilon }\left(i\right)-min\left(P_{\upsilon_{I}}\right)}{max\left(P_{\upsilon_{I}}\right)-min\left(P_{\upsilon_{I}}\right)}\;\;\;\;\forall i\in \phi_{n}$$

A vehicle with the lowest $P_{\upsilon_{norm}}$ has the highest velocity priority.

#### 4.1.4. Overall Election Priority

The overall priority is a function of $P_{e}$, $P_{l}$ and $P_{\upsilon }$ as:

(16) $$P=\frac{1}{w_{1}}P_{e}+\frac{1}{w_{2}}P_{l}+\frac{1}{w_{3}}P_{\nu }$$

$w_{1},w_{2}$ and $w_{3}$ are weighting values used to determine the weight of priority factors (equipment, location and velocity), where $w_{1}+w_{2}+w_{3}=1$.

For one cluster, the overall priority of the vehicles can be obtained by:

(17) $$P_{I}=\left[\begin{array}{ccccc} P_{1} & P_{2} & P_{3} & ... & P_{N_{c}} \end{array}\right]$$

These values can be normalized by scaling them between zero and one as:

(18) $$P_{norm}=\frac{P\left(i\right)-min\left(P_{I}\right)}{max\left(P_{I}\right)-min\left(P_{I}\right)}\;\;\;\;\forall i\in \phi_{n}$$

Usually, $w_{1}$ takes the biggest value because the equipment is quite important for providing reliable service in the network, particularly in intra-cluster communication. The location $\left(w_{2}\right)$ should take the second weight, because the vehicle with the best location has a good possibility to serve the others with the minimum number of hops; consequently, this will reduce the end-to-end delay.

### 4.2. Cluster Head Election Procedure

The CHE procedure is as follows:

1. Each vehicle periodically sends a *HELLO* *(msg, hop-cnt)* message; at the same time, it also forwards the *HELLO* messages of other vehicles. Upon doing *M* hops, the vehicle will be capable of processing all other vehicles’ information, which includes the ID number, the cluster election priority *P*, location information, velocity (υ) and hop count *(hop-cnt)*. Every vehicle upon receiving the *HELLO* message from others stores the other vehicle’s information and subsequently uses this information to determine the hop count *M* of each vehicle and save it for further usage. During the information collection process, the receiving vehicle always determines the hop count of the other vehicle, stores the original *M*, adds one to the count and then forwards the information to the next vehicle. Through this process, the next vehicle will be able to determine the *M* hop of that vehicle. After a number of transmissions, vehicles will have all the neighbors’ information.
2. After information collection, each vehicle (i) calculates P(i) according to Equation (16) and compares the result with P(j), if:
  (19) $$P\left(i\right)<P\left(j\right)\;\;\;\forall j\in \phi_{i}$$
  The vehicle with a lower *P* value has the highest priority, thus it will upgrade itself as a CH. If two or more vehicles share the least value of *P*,
  (20) $$\left\{\begin{array}{c} P(i)=P(j)\\ P_{e}\left(i\right)<P_{e}\left(j\right) \end{array}\right.$$
  The vehicle with the least $P_{e}$ value is selected as a CH, if:
  (21) $$\left\{\begin{array}{c} P_{e}\left(i\right)=P_{e}\left(j\right)\\ ID(i)<ID(j) \end{array}\right.$$
  The vehicle with the least ID should be elected as the CH.
3. The vehicle, after upgrading itself as a CH, will broadcast a message to the outside, propagating cluster information, *HEAD (CluID, head-msg, way-car, hop-cnt)* , where *CluID* is a cluster label, *head-msg* is the CH vehicle information, *way-car* is the route information and *hop-cnt* is the number of hops. In the initial transmission, the value of *way-car* is set to null, and *hop-cnt* is set to zero. Other vehicles within the cluster, upon receiving the *HEAD* message, store all values and examine the value of *hop-cnt*. If *hop-cnt* is less than $M_{max}$, then they will add one to its current value and then forward the information to the next hop. If *hop-cnt* is equal to $M_{max}$, then there is no need for further treatments. Other vehicles when receiving the same *CluID* investigate *M* values and select the lowest value of *M*, then use this value to determine the value of their *hop-cnt* return. When they receive a message from a different *CluID*, they analyze *head-msg* of the CHs, and the information of the high priority vehicle is retained. During the exchange of the HELLO messages, vehicles usually examine the *CluID* when the *hop-cnt* equals one, if a vehicle found that the *CluID* is different from its *CluID*, that means those two vehicles are border nodes.
4. The vehicle sends the apply message *APPLY (CluID, msg, way-car, hop-cnt)* to the CH, which was selected according to Step 2; other vehicles on the route interrupt this message and add their information to *way-car* and add one to the *hop-cnt*, then forward it to the CH.
5. The CH will add the vehicle to its member list just after verifying that the number of vehicles is less than $N_{max}$; in this case, the CH sends back an accept message (*ACCP*) to the intended vehicle; otherwise, it sends back a refuse message (*RFUS*) . If a vehicle receive a *RFUS* message, it will establish a temporary link with one of the neighbor vehicles to access the network resources until the number of vehicles becomes less than $N_{max}$ or joins another cluster.
6. The intended vehicle after receiving the *ACCP* message broadcasts the *MEMR (CluID, msg, way-car, dis-cnt, hop-cnt)* message, and then, there is no need to be involved in the other clustering election process by sending *dis-cnt* to other CHs.
7. Repeat the process until all vehicles become a CH or cluster members.
8. Border cluster members search for the adjacent vehicle of the other cluster using the *hop-cnt* and *CluId* information, making a list of all neighbor nodes.
9. Send a *BUND (msg, way-car, dis-cnt, hop-cnt)* message *hop-cnt* to its CH indicating that it becomes a boundary vehicle.
10. A vehicle with a RFUS message contacts the very near boundary vehicle and sets a temporary connection with it until it finds a new CH or constructs a new cluster with other vehicles.

### 4.3. Cluster Maintenance

After finishing the clustering process and CHE, the vehicles’ movement may change the cluster structure. Therefore, the cluster structure continuously needs maintenance, until the vehicle exits the network. Vehicles periodically check the neighbor list and the neighbors’ cluster listing status, in the case of adding a new cluster member or one of the cluster members leaves its cluster; cluster structure will not change, and only the CH can modify the cluster members list. When a boundary vehicle leaves the cluster, then the CH needs to select a new border vehicle using a similar clustering process and selection criteria. Due to the movement, CHs may move closer to each other, which leads to clusters merging. This situation triggers the CH competition using the same role of the CHE process. The loser CH sends a message to inform its cluster member about the new elected CH. In addition, a new CH should be elected when the loss of the CH occurs due to an accident or any other abnormal situations.

## 5. The Proposed Cluster-Based VANET Routing Protocol

Due to the random node mobility, a major challenge is how to route data packets in a desert and similar scenarios of communication, particularly when the source and the destination are out of the DSRC transmission range. Maintaining a routing table, as in proactive methods, is not an optimal solution, and repetitive path finding before each packet delivery, as in reactive routing, can also be exhaustive. Therefore, specific routing solutions are needed. A routing strategy only based on the location information of the nodes can satisfy the requirements of VANETs in a desert. The problem is that the majority of location-based protocols mainly rely on road side units (RSU), road intersections and other assumptions, which may not be available in a desert. This paper proposes a cluster-based VANET routing protocol (CBVRP). The proposed algorithm covers three communication scenarios, as follows.

### 5.1. Routing within a Cluster

When a cluster member needs to establish a link, it sends a request to the CH. The CH after receiving the request verifies whether the intended vehicle is a member of the cluster or not. In case both vehicles are in the same cluster, the CH finds from its storage the location information of the source and the destination vehicle, then starts the process of best route selection depending on the destination and source locations.

**Figure 6:**
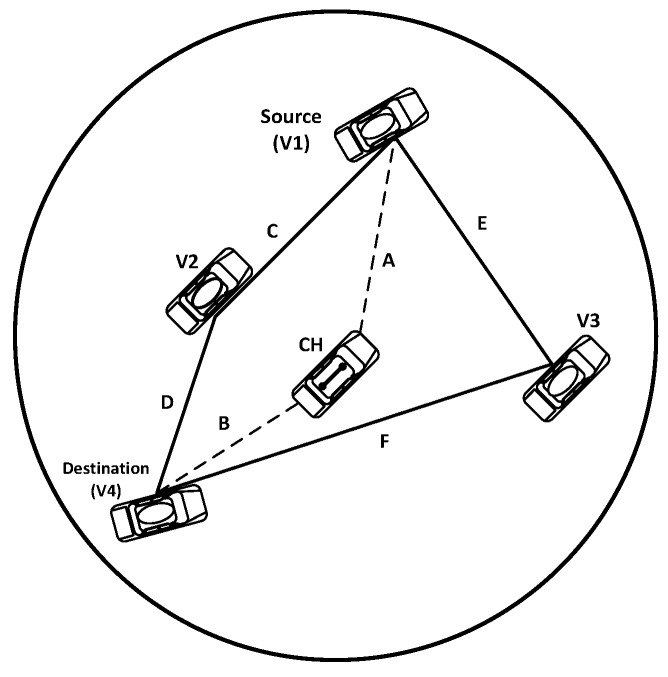
Routing within a cluster.

For better understanding of the proposed inter-cluster routing protocol, we assume five vehicles, as follows. $V_{1}$ is the source vehicle located at the coordinates $\left(x_{V_{1}},y_{V_{1}}\right)$. Both $V_{2}$ and $V_{3}$ are two vehicles in between the source and the destination with $\left(x_{V_{2}},y_{V_{2}}\right)$ and $\left(x_{V_{3}},y_{V_{3}}\right)$ coordinates, respectively. $V_{4}$ is the destination vehicle located at $\left(x_{V_{4}},y_{V_{4}}\right)$ coordinates, as shown in Figure 6.

The distance between the CH to $V_{1}$, $V_{2}$, $V_{3}$ and $V_{4}$ satisfies Equation (22). The CH selects the best route according to:

(22) $$L\geq \sqrt{{\left(x_{i}-x_{j}\right)}^{2}+{\left(y_{i}-y_{j}\right)}^{2}}$$

(23) $$A\geq \sqrt{{\left(x_{ch}-x_{V_{1}}\right)}^{2}+{\left(y_{ch}-y_{V_{1}}\right)}^{2}}$$

(24) $$B\geq \sqrt{{\left(x_{ch}-x_{V_{4}}\right)}^{2}+{\left(y_{ch}-y_{V_{4}}\right)}^{2}}$$

(25) $$C\geq \sqrt{{\left(x_{V_{1}}-x_{V_{2}}\right)}^{2}+{\left(y_{V_{1}}-y_{V_{2}}\right)}^{2}}$$

(26) $$D\geq \sqrt{{\left(x_{V_{2}}-x_{V_{4}}\right)}^{2}+{\left(y_{V_{2}}-y_{V_{4}}\right)}^{2}}$$

(27) $$E\geq \sqrt{{\left(x_{V_{1}}-x_{V_{3}}\right)}^{2}+{\left(y_{V_{1}}-y_{V_{3}}\right)}^{2}}$$

(28) $$F\geq \sqrt{{\left(x_{V_{3}}-x_{V_{4}}\right)}^{2}+{\left(y_{V_{3}}-y_{V_{4}}\right)}^{2}}$$

**Figure 7:**
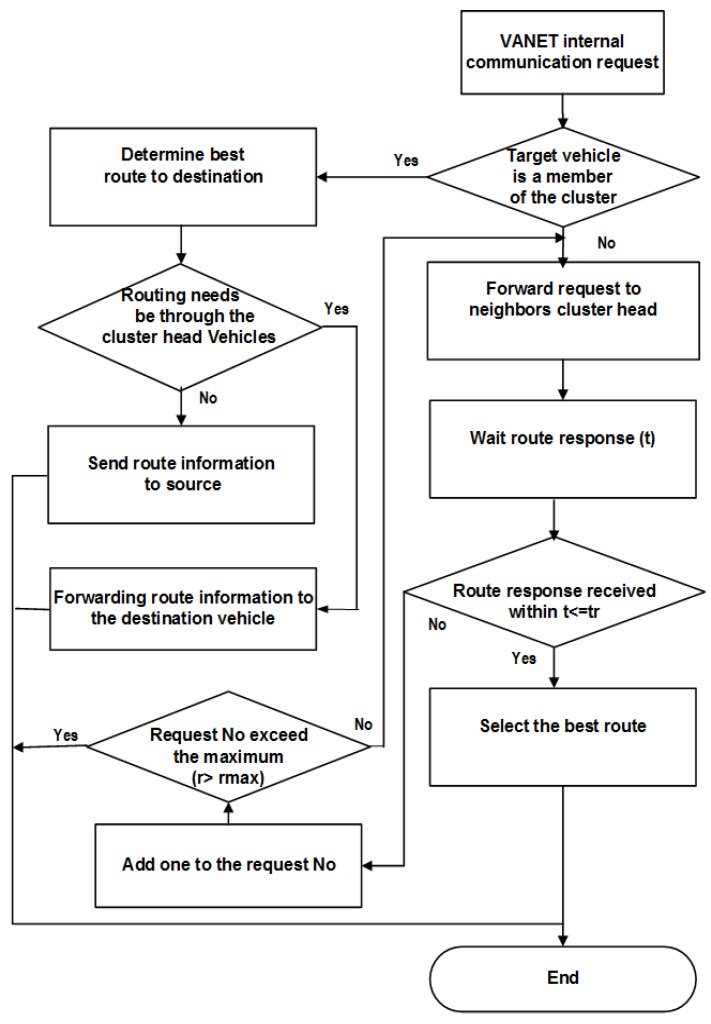
Routing algorithm within a cluster.

If C+D≤A+B, hence, the route C,D is the best one, and the next hop is $V_{2}$. The CH notifies $V_{1}$ about the best route, which decreases the risk and burden on the CH. If A+B<C+D, this means that the best route will be through the CH, which will forward the packet to $V_{4}$. That is to say, besides being responsible for the route selection, the CH may also participate in the packet forwarding process. The route E,F will be stored as a backup. The overall procedure flowchart is described in Figure 7.

### 5.2. Routing between Clusters

**Figure 8:**
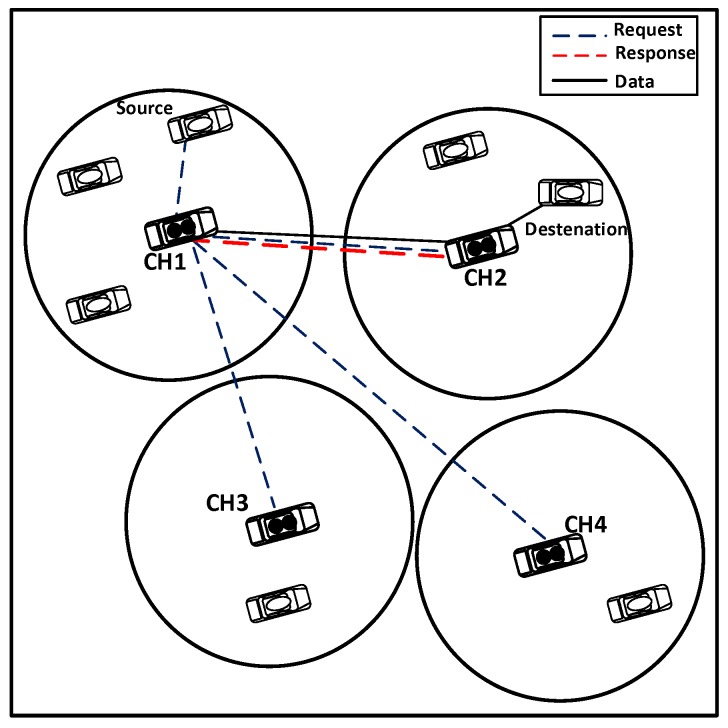
Routing between clusters.

When the CH does not find the needed information in its internal storage, it requests the closest CHs for the destination information and waits for a routing response. If the waiting time exceeds the threshold $t_{r}$ and the route response has not yet been received, re-request message (*RREQ*) is resent. If the retransmission exceeds the maximum retransmission limit $\left(r_{max}\right)$, the route search process is terminated. To reduce network congestion, not all of the neighboring CHs receiving *RREQ* will respond. Only those located on the route towards the destination having the ability to serve will participate in the routing process. As shown in Figure 8, only $CH_{1}$ and $CH_{2}$ participate in the routing process.

Therefore, neighbor CHs, after having received the *RREQ* message, first determine if they are located on the route of the CH request; if not, the request is discarded. Otherwise, the procedure is as follows.

**Figure 9:**
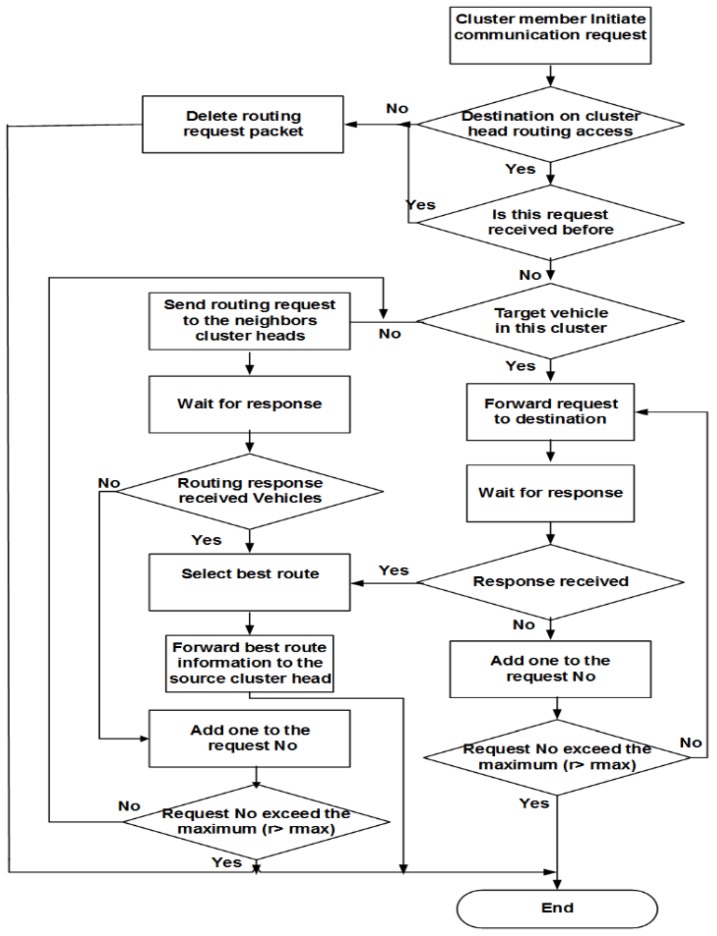
Routing algorithm for public networks and between clusters.

1. A neighboring CH checks the request; if it is the first time received, then it continues the procedure; if not, then the request is discarded.
2. The neighboring CH checks whether the destination vehicle is in its cluster or not. If not, then apply Step 4.
3. The neighboring CH forwards a route request to the intended vehicle and waits for vehicle routing response *RREP*; also, go to Step 5.
4. The CH adds its own *msg* to the (*REEQ msg*) message, and then forwards to the next hop neighbors’ CH and waits for the route response.
5. If the waiting time exceeds $t_{r}$, and it does not receive the route response, then retransmit the request and add one to request number; otherwise, go to Step 7.
6. If the number of retransmissions exceeds the limit $r_{max}$, then end the route request process; alternatively, in accordance with the intended vehicle situation, directly apply Step 3 or Step 4.
7. If the CH receives more than one route response, then it chooses the vehicle with the fewest numbers of routing hops and the minimum distance to the destination and adds its own *msg* to the (*REEP msg*) message; then, it forwards this message to the previous hop CH vehicle at the same time, saving other routes as backups. If the routing request fails, probably due to the destination cluster, which is far away from the source cluster, and the routing communication request cannot be established through the VANET or through neighbor vehicles, the CH may establish a connection via satellite or mobile communication.
8. In some situations and according to the CHE process, the CH may have no satellite or mobile equipment to communicate with the far away CHs; in this case, the CH searches for one of its cluster members, which is equipped with satellite or mobile communication, to forward the request to the neighboring CHs.
9. If still not be able to establish the connection and routing through satellite or mobile communication, then it sends the notification *REER* to the source vehicle, indicating routing failure. The flowchart of the procedure of routing between vehicles located in different clusters is shown in Figure 9.

### 5.3. Cluster Member’s Communication with the Public Networks

**Figure 10:**
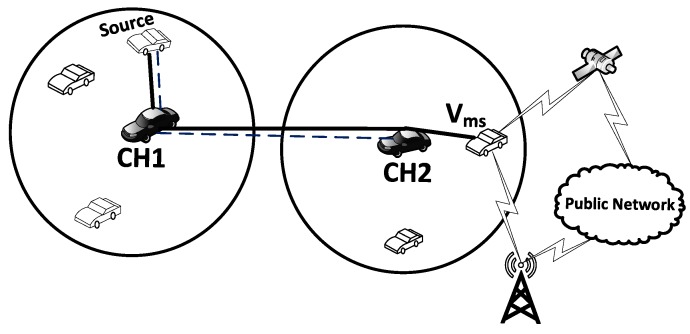
Communication to the public network using cluster member type $V_{ms}$.

In normal situations, only vehicle types $V_{m}$, $V_{s}$ and $V_{ms}$ can communicate with the outside networks. Vehicles always prefer using the VANET, but if the destination is out of the VANET coverage or they need to communicate with public networks and the destination is unreachable through the multi-hop VANET, in such cases, the source CH firstly searches its cluster members to find a member that is equipped with an appropriate communication link to communicate with the public network. If no cluster members are equipped with suitable equipment to communicate with the public network, then it forwards the request to the neighboring CH, which is equipped with the appropriate communication link, as shown in Figure 10. Neighboring CHs upon receiving the communication request will proceed as follows.

1. Check the request. If this is the first time receiving, it continues the procedure; if not, the request will be discarded.
2. Check whether its equipment is type *V*, and then, it will skip to Step 5.
3. If the vehicle is type $V_{m}$, then it will skip to Step 9.
4. Check whether this cluster has a free available $V_{m}$ or $V_{ms}$, then it forwards the routing request and waits for a response, then skips to Step 6.
5. Forward the routing request to the neighboring CH, and wait for a response.
6. If the waiting time exceeds $t_{r}$ and it does not receive the route response, then it retransmits the request and adds one to the request number; otherwise, skip to Step 8.
7. If the number of retransmissions exceeds the limit $r_{max}$, then end the route request process; in the case of vehicle $V_{m}$, return to Step 4, while for $V_{ms}$, return to Step 5.
8. If the CH receives more than one route response, it will choose the vehicle with the fewest number of routing hops, the minimum cost and the shortest distance to the destination. At the same time, it stores the other routes as backup routes.
9. It Adds its own *msg* to the (*REEP msg*) message and forwards the message to the previous hop CH in the source direction. If the routing process fails to find a vehicle type $V_{m}$, the source vehicle searches for a vehicle type $V_{s}$ using a similar way as mentioned in the above steps. If it is still not able to establish a route through a satellite or mobile connection, it will send a *REER* notification to the source vehicle indicating a routing failure.

### 5.4. Route Maintenance

Due to vehicle’s movement, the established route may be lost. Consequently, the communication will temporarily disconnect. At the same time, the vehicle will store the destination routing information for a while and try to resend the request. If it succeeds in sending a new route request to the intended destination, that means the route has been recovered. If it fails to send the request, then it will search its backup routing information and try to use one of the best backup routes. If it fails to send the message, it will start a new route finding procedure.

## 6. Simulation and Results

### 6.1. Simulation Setups and Configurations

We implemented a simulation to demonstrate the velocity, equipment, location and movement direction of the vehicles. The purpose behind the simulation is to evaluate the CHE process. The simulation firstly assumes a number of vehicles randomly moving in a specific area and uses the C4.5 algorithm to classify them according to the direction. The C4.5 algorithm classifies the vehicles into four groups according to their directions. Then, for any group moving in the same direction, the simulation performs the clustering process and consequently calculates the CH. The vehicles in one cluster have different values of velocity, location and equipment. We assumed that the velocity and location of all vehicles are normally distributed. For the simulation purposes, we suppose that the number of vehicle type $V_{ms}$, $V_{s}$, $V_{m}$ is 5%, 10%, 15%, respectively. In the simulation, we used $w_{1}=0.5$, $w_{2}=0.3$ and $w_{3}=0.2$. The main parameters used for this simulation are listed in Table 2.

The simulation was executed several times with different numbers of vehicles to calculate the different values of the priority factors. In the first part of the simulation, the priority factors *versus* the ID numbers of the vehicles are plotted based on the different values.

Since the elected CH plays a major role during the routing process, we used the same scenario to evaluate the proposed routing algorithm. The system uses IEEE802.11p technology specifications during the simulation. We evaluated CBVRP by comparing it to the AODV, CBRP and DSR routing protocols [12,13]. DSR is selected because of its dynamic characteristics, and CBRP is selected with the aim to compare the CBVRP with one that has the same clustering properties, while the AODV protocol is chosen, because it has the on-demand property. Besides, these protocols are also considered as the latest VANET routing protocols.

The performance of the CBVRP is evaluated against AODV, DSR and CBRP in terms of cluster structure reconstructions *versus* time, packet delivery ratio (PDR), average end-to-end delay (AD) and routing cost. The PDR is defined as the percentage of packets that is successfully received by the destination nodes to the packets sent by source nodes and can be calculated according to Equation (29).

(29) $$PDR=\frac{1}{m}\sum_{i=1}^{m}\frac{Rx_{i}}{Sx_{i}}$$

where *m* here is the total number of connections, $R_{x}$ is the total number of successfully received packets for one node, $S_{x}$ is the total number of packets that have been sent to the same node and *i* is an index value representing the ID of the designated connection. AD is defined as the average time between a packet being sent and being received and can be calculated according to Equation (30).

(30) $$AD=\frac{1}{Rx_{i}}\sum_{j=1}^{Rx_{i}}\left(tr_{j}-ts_{j}\right)$$

where *j* here is a packet identifier, $tr_{j}$ is the time at which a packet *j* is received and $ts_{j}$ is the time at which a packet *j* is sent [26].

### 6.2. Results and Discussion

During simulation, we realized that the main problem of the three protocols is that they have a limitation when used in desert environments to connect the source and destination located in different clusters, and there is no direct connection between them. As these protocols are originally designed for MANETs rather than VANETs, they have been utilized in VANETs in urban and highways scenarios. Moreover, deserts have no RSU to support intra-cluster communications. CBVRP bridges the gap, because it has been particularly designed for such environments. However, it would be also acceptable for any area to allow communication between randomly moving vehicles. Simulation results are shown in the following figures. The graphs show the election procedure result and comparison between the three mentioned protocols by using different parameter values as the basis of the above-mentioned metrics.

**Figure 11:**
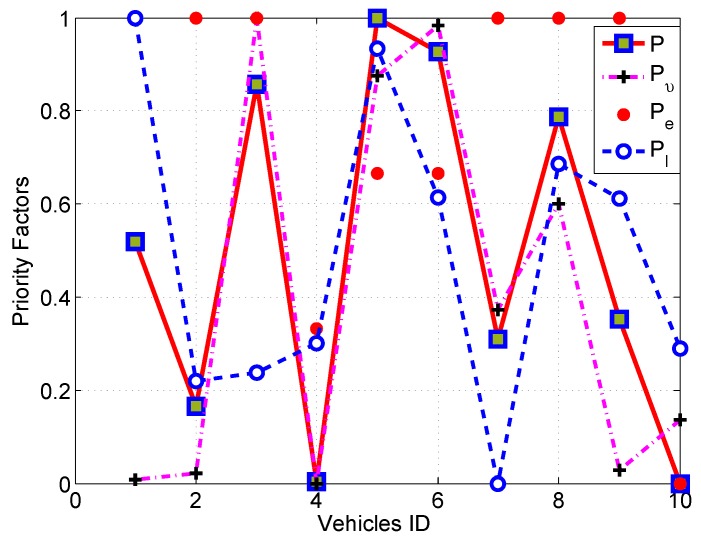
VANET cluster head election (CHE) priority factors.

Figure 11 shows all priority factors (*P*, $P_{e}$, $P_{l}$ and $P_{\upsilon }$) with respect to the vehicle’s IDs. According to the *P* value, vehicle ID Number 10 is elected as a CH. To elect the CH using only the velocity, as other routing protocols do, vehicle ID Number 1 will be elected, although it is equipped with VANET only and the location of it is quite worse. In addition, in the case of using the location, vehicle ID Number 7 has a high location priority, but its equipment and velocity are worse. The use of overall priority will solve the problem by using a weighting mechanism in order to achieve a better utilization of all vehicles and the optimum election. This problem has not been addressed by other existing protocols, which use location or velocity to elect the CH. Moreover, as can be seen from Figure 11, vehicles with ID Numbers 4 and 10 have the same overall priority factor. In this case, the vehicle with the best equipment will be elected as a CH, as specified in Equation (20).

**Figure 12:**
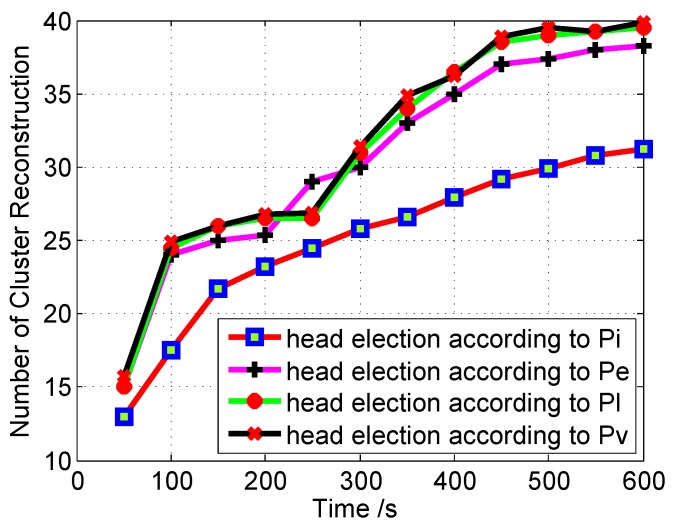
Cluster reconstruction *versus* time; here, the CH is elected according to different election priority factors.

Figure 12 demonstrates the effect of CHE on the stability of the cluster structure when using different types of priority factors.

The effect of $P_{l}$ and $P_{v}$ seems to be the same, because the location of vehicles is affected by the velocity. The variation in the velocity of the well-equipped vehicles may affect its overall priority. When *P* is used, the cluster structure is more stable, because the selection process considers all priority factors.

**Figure 13:**
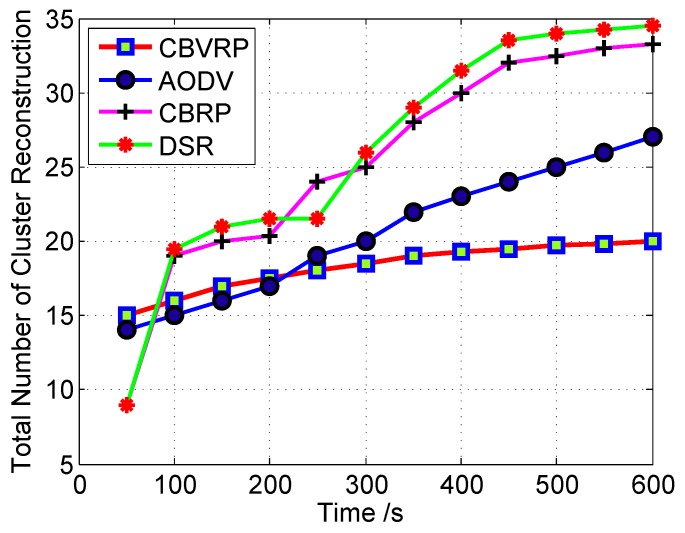
Cluster structure stability over time for cluster-based VANET routing protocol (CBVRP), CBRP and dedicated short-range (DSR) protocols.

Figure 13 shows that the cluster structure stability of CBRP and DSR is poor. This is because the CBRP algorithm uses only the ID of the vehicle as a basis for CHE. The smallest ID is elected as a CH. A cluster structure may change rapidly in the case of a vehicle moving with high velocity. In case of DSR, a separate periodic algorithm must be implemented to support and propagate the CH advertisements across the cluster. The response of the algorithm may affect the cluster structure.

The CBVRP CHE process takes into consideration the movement of neighboring vehicles in addition to location and equipment, to reduce the probability of cluster change over time, to have a higher stability.

**Figure 14:**
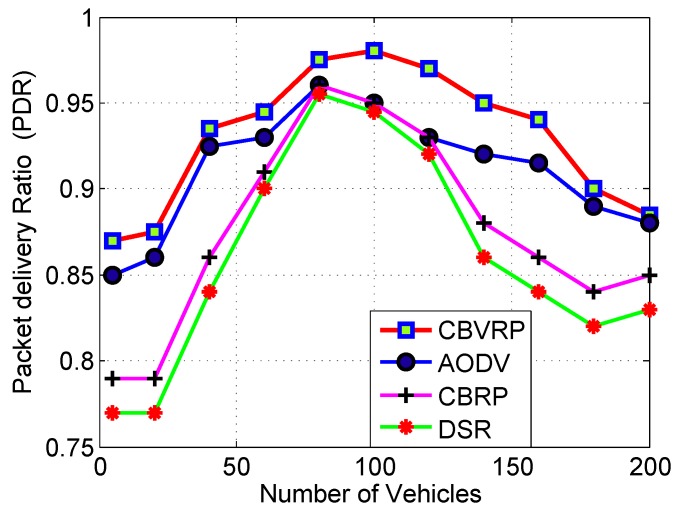
PDR *versus* numbers of vehicles for CBVRP, CBRP, DSR.

Figure 14 shows comparison between CBVRP, AODV, CBRP and DSR protocols in terms of PDR. It is observed that PDR of CBVRP remains high while increasing the number of vehicles. The increase of the number of vehicles did not affect the PDR because of the high efficiency and cluster structure stability of the routing algorithm. The PDR of CBRP and DSR is less than that obtained by CBVRP and AODV, because the source node and the intermediate nodes store the next hop information corresponding to each flow for data packet transmission, but DSR and CBRP use source routing in which a data packet carries the complete path to be traversed. CBVRP outperforms AODV because of the store route as backup and built-in route maintenance (RM) properties, while in the case of AODV, the routing mechanism searches for a new route at every request and when a route failure has occurred. The use of RM increases the packet delivery ratio, saves route rediscovery flooding traffic and reduces overall route acquisition delay. The PDR is too low in the beginning of the curves due to the random initiation of the simulation program.

**Figure 15:**
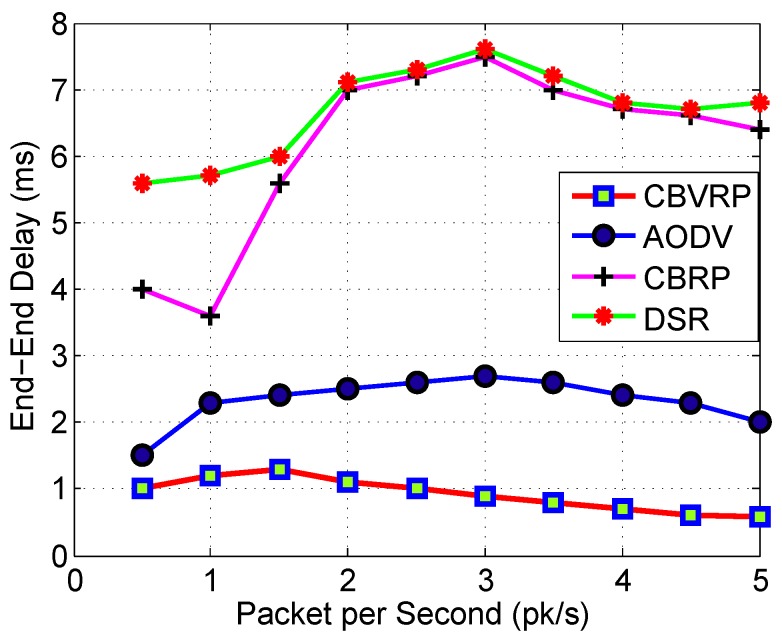
CBVRP, CBRP and DSR number of sent packet *versus* end-to-end delay for 20 vehicles.

Figure 15 shows a number of sent packets *versus* end-to-end delay for CBVRP, AODV, CBRP and DSR. In the figure, we can observe that the average end-to-end delay of CBVRP is the best among the others. The packet delivery delay time is affected by the route search algorithm and the packet delivery process itself. CBVRP end-to-end delay is better than that of others because the CBVRP route search algorithm is done only once and remains stable until the cluster structure changes. This decreases the time needed for the whole process of packet delivery. AODV requires more time to establish a connection, and the initial communication required for finding a route is dense; however, it has no extra traffic for communication along existing links. For that reason, it has more advantage over DSR and CBRP, and its AD is less than that of DSR and CBRP; moreover, its AD decreases with time. Other protocols had a longer delay because the route finding process takes more time, as every intermediate node tries to extract information before forwarding the reply, while the protocol tries to search for a new route.

**Figure 16:**
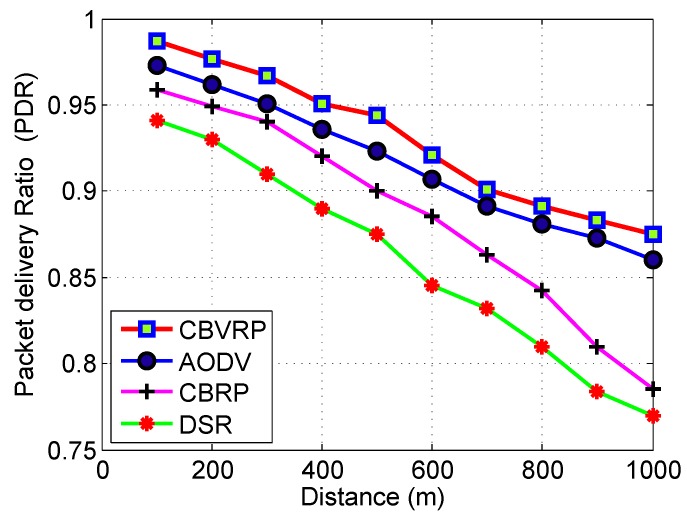
The PDR of CBVRP, CBRP, AODV and DSR *versus* the communication distance.

**Figure 17:**
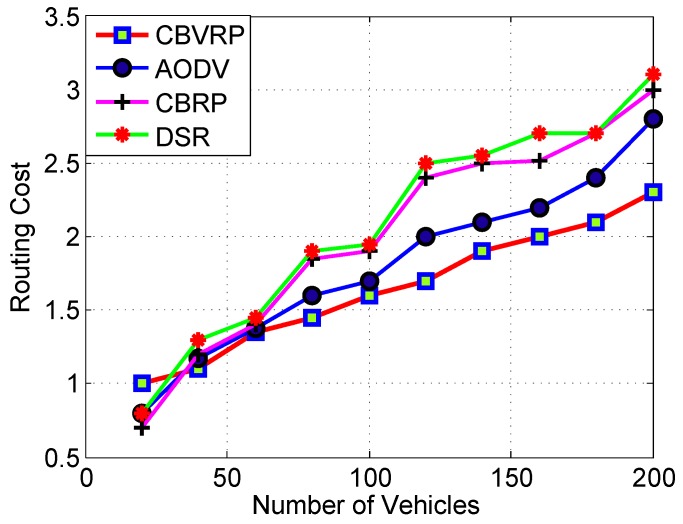
CBVRP, CBRP, AODV and DSR routing cost *versus* the number of vehicles.

We also investigated the effect of the communication distance on the performance of the proposed routing protocol, and we compared it to AODV, CBRP and DSR. The results are shown in Figure 16. As is clear from the figure, the PDR performance deteriorated with the increase of distance. Meanwhile, the proposed protocol demonstrated a superior performance compared to the others. This result clearly indicates the suitability of this protocol for desert and similar scenarios. According to Figure 17, we noticed that the routing cost of CBVRP, AODV, CBRP and DSR increases with respect to the increase of the number of vehicles; this is because the increase in the number of vehicles directly increases the number of hops, thereby increasing congestion in the routing process. In the case of fewer vehicles, the CBVRP routing cost is more than CBRP and DSR, because in CBVRP, the initiation of the route finding process is very complicated. It takes more time in the initialization of the process. However, this calculation is done once and remains steady until the cluster structure changes. Hopefully, the increase in the number of vehicles will increase the opportunity of more backup routes in CBVRP, which enhances the route maintenance process. The AODV problem is that the increase of the intermediate nodes can lead to inconsistent routes when the source sequence number is very old and the intermediate nodes have a higher, but not the latest destination sequence number, thereby having none existent or stale entries. In the case of more vehicles, CBRP and DSR have a higher routing cost, because they use a flooding approach with all of the vehicles to determine the destination route, while in CBRVP, only vehicles in the request cluster that have the capability of finding the destination are able to participate in the routing process.

## 7. Conclusions

This paper presented a novel clustering-based VANET routing algorithm protocol (CBVRP) appropriate for desert scenarios. CBVRP is mainly based on a vehicle’s equipment, location and velocity, which play a major role in cluster classifications and CHE. A stable clustering approach is used to reduce on-demand routing. Furthermore, a local route maintenance mechanism was utilized to reduce the delay caused by the new route finding process. The routing algorithm approach proposes that when communication is within a single cluster, the CH directly selects the most appropriate vehicle to serve as the next hop in the route to the destination. In order to communicate with the outside, the CH uses flooding methods towards its cluster members, seeking a route using the nearest suitable vehicles able to communicate with the outside. This route remains stable until the cluster structure changes. Other available routes are stored as backup. CBVRP is evaluated using simulation by comparing it to other alternatives and against other protocols. Evaluation results show that the proposed algorithm characteristics have high efficiency and scalability. A VANET using CBVRP obtains higher stability, significant success rates of data transfer, lower routing cost and decreased packet transmission delay. Further research can focus on studying the capability of using CBVRP bidirectional routing, considering security issues and enhancing the CHE process by adding more priority factors, such as signal strength.

## Figures and Tables

**Table 1:** Simple training set for vehicles’ direction classification used by C4.5.

Orientation	Class
$316^{\circ }$ : $45^{\circ }$	East
$46^{\circ }$ : $135^{\circ }$	North
$136^{\circ }$ : $225^{\circ }$	West
$226^{\circ }$ : $315^{\circ }$	South

**Table 2:** Parameter values of the simulation.

Parameter	Value	Parameter	Value
Network area	5000 × 5000 m	Data rate b/s	512 kb/s
Vehicle coverage distance (*L*)	1000 m	Time-To-Live (TTL) (k)	8
Velocity range	0 to 120 km/h	$r_{max}$	4
Total number of vehicles (*N*)	20 to 200	$t_{r}$	8 s
Max number of vehicles per cluster $\left(N_{max}\right)$	5 to 20	Simulation time	480 s
